# Plasma Gamma Globulin Levels After Splenectomy and Spleen Salvage

**DOI:** 10.1155/1989/67203

**Published:** 1989

**Authors:** Yesilkaya Yasar, Yilmaz Zeki, Erkilic Ahmet, Sen Metin, Kilic Hüseyin

**Affiliations:** Department of Surgery and Microbiology Erciyes University School of Medicine Kayseri Turkey

## Abstract

A series of plasma globulin studies was carried out on 108 patients who were operated on for splenic trauma during the last 3 years. The reasons for splenectomy or spleen salvage were; gunshot wounds in 22 patients (20.3%); stab injuries in 10 patients (9.2%) and blunt abdominal trauma in 76 patients (70.3%). Plasma gamma globulin determinations were made on the 8th postoperative day and at 3 months. In the splenectomy group; plasma gamma globulin determinations demonstrated a significant reduction in serum IgM levels (p < 0.001) but no significant changes in IgA and IgG levels (p > 0.05). No changes were detected in IgA, IgG and IgM levels in the spleen salvage group (p > 0.05).

We believe that the preservation of the traumatized spleen should be the prime aim of surgeons.


*HPB Surgery* 1989, Vol. 1, pp. 97–100
Reprints available directly from the publisher
Photocopying permitted by license only

© 1989 Harwood Academic Publishers GmbH
Printed in Great Britain

### PLASMA GAMMA GLOBULIN LEVELS AFTER SPLENECTOMY AND SPLEEN SALVAGE

YESILKAYA YASAR*, YILMAZ ZEKI, ERKILIC AHMET, SEN METIN and KILIC HÜSEYIN

*Department of Surgery and Microbiology, Erciyes University, School of Medicine, Kayseri, Turkey*

*(Received 16 March 1988; in final form 8 June 1988)*

A series of plasma globulin studies was carried out on 108 patients who were operated on for splenic trauma during the last 3 years. The reasons for splenectomy or spleen salvage were; gunshot wounds in 22 patients (20.3%); stab injuries in 10 patients (9.2%) and blunt abdominal trauma in 76 patients (70.3%). Plasma gamma globulin determinations were made on the 8th postoperative day and at 3 months. In the splenectomy group; plasma gamma globulin determinations demonstrated a significant reduction in serum IgM levels ($p < 0.001$) but no significant changes in IgA and IgG levels ($p > 0.05$). No changes were detected in IgA, IgG and IgM levels in the spleen salvage group ($p > 0.05$).

We believe that the preservation of the traumatized spleen should be the prime aim of surgeons.

KEY WORDS: Splenectomy, splenorrhaphy, abdominal trauma, plasma gamma globulins

### INTRODUCTION

King and Shumacher[1] in 1952 were the first to point out the serious effects of infection in splenectomized patients. This was later confirmed by a number of investigators. In view of the role of the spleen in immune response and defence against infection, reassesement of our attitude toward splenectomy is mandatory[2,3,4,5,6].

Splenectomy is associated with a number of alterations in host defences, which may explain the increased susceptibility to bacterial infections that occurs as a consequence of this procedure. Decreased properdin levels and diminished alternative complement pathway activation have been reported in splenectomized patients[3,4]. The human spleen is also the production site of a peptide that promotes phagocytosis[6]. As a consequence of these immunological alterations, post-splenectomy sepsis may develop with a mortality rate which may be as high as 50%. Neither age nor the length of time following splenectomy provides absolute protection[4,7].

We have planned this study prospectively to demonstrate any difference in plasma gamma globulin levels after splenectomy or spleen salvage.

### METHODS

During the last 3 years, 108 patients with splenic injury were identified during laparotomy at Erciyes University Hospital. Splenectomy for Haemotological

*Correspondence to: Prof. Dr. Yasar Yesilkaya, Director of Surgery, Erciyes University, Kayseri, Turkey.

indications and iatrogenic splenic injuries have not been included in this series. Age at the time of operation ranged from 1 to 67 with a mean age of 17.8 years. Twenty eight of the patients were female (25.9%) and 80 were male (74.1%). 22 patients (20.3%) had gunshot wounds, 10 patients (9.2%) had stab injuries and 76 patients (70.3%) had blunt abdominal trauma.

We divided the patients with splenic injuries into 2 groups: splenectomy (Group-A) and spleen salvage (Group-B). Splenectomy was performed on 60 of 108 patients (55.5%) and spleen salvage in 48 of 108 patients (44.4%). The type of injury was determined at laparotory (Table 1). Depending on the type of the splenic injury, general condition of the patient and presence of any associated non-splenic injuries, splenectomy or spleen salvage was performed. The techniques employed related to the injury types are shown in Table 2. Spleen salvage was accomplished by means of a hot compress only in type 1 and splenorrhaphy in type 2 and 3. Three patients in type 3 underwent a partial splenectomy of 10–30%, but they were excluded from the study. Splenorrhaphy was by means of 3/0 absorbable figure of eight sutures.

All patients were observed for a period of at least 3 months following the operation. A set of plasma gamma globulin determinations was performed on all patients on the 8th postoperative day and after 3 months. The determination the levels of gamma globulin A,G and M were carried out in agar gel in "Behringwerke" plates by the single radial immuno-diffusion technique described by Mancini *et al*[8].

Splenectomy and spleen salvage groups were compared with each other for plasma globulin alterations. The data was analysed by Student's T-test.

**Table 1** Types and Definitions of splenic injuries

| *Type of injury* | *Definition of type* |
|---|---|
| Type 1 | Capsular tear without parenchymal injury. |
| Type 2 | Capsular tear with parenchymal injury which does not extend to the splenic hilum. |
| Type 3 | Deep laceration extending to the splenic hilum. |
| Type 4 | Completely shattered or avulsed spleen. |

### RESULTS

In the splenectomy group, the mean gamma globuilin M level was 211.5±59.5 mg/dl on the 8th postoperative day, and 105.7±51.2 mg/dl in the 3rd postoperative month. This is a considerable decrease in gamma globulin M levels ($p < 0.001$). There were statistically no significant changes in levels of gamma globulin A or G between the 8th day and 3rd month ($p > 0.05$) (Table 3).

In the spleen salvage group, no change was observed in the gamma globulin A,G or M levels between the 2 determinants ($p > 0.05$) (Table 3).

While there were neither any deaths nor complications in patients following the splenic salvage, there were two deaths (3.33%), one as a result of closed head injury and the other related to postoperative pancreatitis, and 10 complications in the splenectomy group. These deaths and complications were related to the patients serious general condition, and the severity and the number of their associated non-splenic injuries. The number of non-splenic injuries was 77 in the

**Table 2** The techniques employed according to the injury types

| *Types of injury* | *Splenectomy group Number of Patients* | *Spleen salvage group Number of Patients* |
|---|---|---|
| Type 1 | – | 8 |
| Type 2 | 3 | 11 |
| Type 3 | 18 | 29 |
| Type 4 | 39 | – |
| Total | 60 | 48 |

**Table 3** Gamma globulin A,G and M levels (mg/dl) after splenectomy (Group-A) and Spleen salvage (Group-B) on the 8th postoperative day in the 3rd month.

| | | *Postoperative 8th day* | *Postoperative 3rd month* |
|---|---|---|---|
| | | *Mean SD* | *Mean SD* |
| IgA | Group-A | 245.5±78.1 | 253.8±82.5 |
| | Group-B | 229.3±79.8 | 220.6±89.7 |
| IgG | Group-A | 1680.5±275.4 | 1630.2±355.5 |
| | Group-B | 1608.8±519.2 | 1589.1±389.6 |
| IgM | Group-A | 211.5±59.5 | 105.7±51.2 |
| | Group-B | 289.7±91.2 | 269.7±72.7 |

splenectomy group but only 36 in the spleen salvage group. Furthermore, there were altogether 27 isolated splenic injuries, 18 of which were in spleen salvage and 9 in splenectomy group.

### DISCUSSION

The spleen is a lymphoid organ representing 25% of the reticuloendothelial system and known to be an important component of host defences. Acting as an immunological filter, it clears particulate antigens in the circulating blood. The spleen can elaborate and initiate the immune response, stimulating the manufacture of immunoglobulin M antibodies against circulating bacterial antigens. It produces opsonins such as "Tuftsin" that promotes phagocytosis of particulate matter, bacteria and aged red cells. It is also an important organ in the regulation of both T and B lymphocytes[3,4,5,6,9,10].

The immune aberration following splenectomy includes decreased serum immunoglobulin M levels, altered serum opsonic function and failure to respond to intravenous particulate matter. The risk of overwhelming postsplenectomy infection in individuals having a splenectomy for trauma is 50 fold greater than that of the normal population[4].

Overwhelming infection after splenectomy is now an accepted fact. The spleen itself appears to provide a protective function against bacteremia, particularly due to pneumococcus. This protection may be in the form of a mechanical filter, because of its anatomical position and microstructure, in addition to its role as an immunological organ, producing both antibodies and other undefined protective substances[2,3,4,5].

We consider that all of our post-splenectomy patients are in danger of fatal sepsis. However, the most important factors for deciding on splenectomy or spleen salvage, are the operative findings and the surgeon's decision. The splenic vascularity and the amount of retained tissue mass are important for protection from post-splenectomy sepsis[11]. We conclude that the decision of choice, if possible, is the preservation of vascularity and simple repair of rupture or partial splenectomy.

### *References*

1. King, H., Shumacher, H.B. (1952) Splenic studies 1. Susceptibility to infection after splenectomy performed in infancy. *Annals of Surgery*, **136**, 239–242.
2. Balfanz, J.R., Nesbit, M.E., Jarvis, C., Krivit, W. (1976) Overwhelming sepsis following splenectomy for trauma. *Journal of Pediatrics*, **88**, 458–460.
3. Claret, I., Morales, L., Montoner, A. (1975) Immunological studies in the postsplenectomy syndrome. *Journal of Pediatric Surgery*, **10**, 59–64.
4. Krivit, W., Giebnik, G.S. Leonard, A. (1979) Overwhelming postsplenectomy infections. *The Surgical Clinics of North America*, **59**, 223–233.
5. Posey, D.L., Marks, C. (1983) Overwhelming postsplenectomy sepsis in childhood. *The American Journal of Surgery*, **145**, 318–321.
6. Shumacher, M.J. (1970) Serum immunoglobulin and transferrin levels after childhood splenectomy. *Archives of Disease in Childhood*, **45**, 114–117.
7. Barrett, J., Shcaft, C., Abuabara, S., Jonasson, O. (1983) Splenic preservation in adult blunt and penetrating trauma. *The American Journal of Surgery*, **145**, 313–317.
8. Mancini, G., Carbonara, A.O., and Heremans, J.F. (1965) Immunological quantitation of antigens by simple radial immunodiffusion. *Immunochemistry*, **2**, 235–238.
9. Buntain, W.L., Lynn, H.B. (1979) Splenorrhapy: Changing concepts for the traumatized spleen. *Surgery*, **86**, 748–760.
10. Morganstern, L. (1979) Techniques of splenic concervation Archives of Surgery, **144**, 449–454.
11. Millikan, J.S., Moore, E.E., Moore, G.E., Stevans, R.E. (1982) Alternatives to splenectomy in adults trauma of splenic tissue. *The American Journal of Surgery*, **144**, 711–716.

*Accepted by S. Bengmark on 8 June 1988.*

